# Development of a novel patient-reported measure for acromegaly: the Acro-TSQ

**DOI:** 10.1007/s11102-019-00986-4

**Published:** 2019-09-14

**Authors:** Maria Fleseriu, Leon Fogelfeld, Murray B. Gordon, Jill Sisco, Hilary H. Colwell, William H. Ludlam, Asi Haviv, Susan D. Mathias

**Affiliations:** 1 Departments of Neurosurgery and Medicine, Northwest Pituitary Center, Oregon Health and Science University, 3303 SW Bond Ave, CH8N, Portland, OR 97239 USA; 2 John H. Stroger Jr. Hospital of Cook County, Rush University Medical Center, Chicago, IL USA; 3 Allegheny Neuroendocrinology Center, Allegheny General Hospital, Pittsburgh, PA USA; 4 Acromegaly Community, Grove, OK USA; 5 Health Outcomes Solutions, Winter Park, FL USA; 6 Chiasma, Inc, Waltham, MA USA

**Keywords:** Acromegaly, Patient reported outcomes, Quality of life, Acro-TSQ, Questionnaire

## Abstract

**Purpose:**

Somatostatin analogs (SSAs) represent a mainstay of medical treatment for acromegaly, currently available as either intramuscular or deep subcutaneous injections. Patient-reported outcomes (PROs) are increasingly common as relevant outcomes in studies of acromegaly and its treatment, but there are no validated PRO measures available that focus on the disease burden and the impact of treatment, specifically designed for use in patients with acromegaly. We sought to develop a new and unique PRO measure, the Acromegaly Treatment Satisfaction Questionnaire (Acro-TSQ).

**Methods:**

Concept elicitation (CE) interviews were conducted with acromegaly patients in the United States receiving SSA injections at a stable dose for ≥ 6 months. A questionnaire was drafted based on these interviews; combined CE and cognitive debriefing (CE/CD) interviews were then conducted to confirm the content, clarity, and relevance of the questionnaire.

**Results:**

Nineteen subjects completed interviews [n = 9 CE, n = 10 CE/CD; n = 15 Lanreotide Depot/Autogel (Somatuline), n = 4 Octreotide LAR (Sandostatin LAR)]. Most subjects responded positively when asked about the effectiveness of their current treatment; however, breakthrough symptoms, injection site reactions, and side effects were commonly reported and had negative impacts on social and emotional well-being and daily activities. All 10 subjects involved in debriefing interviews found the questionnaire to be relevant, easy to complete, and found the response options to be clear. The resulting 26-item Acro-TSQ covers symptoms and symptom control, gastrointestinal side effects and their impact on daily activities, the emotional impact of treatment, convenience and ease of use, and overall satisfaction.

**Conclusions:**

The Acro-TSQ is a novel PRO, focused on both disease burden and impact of treatment; it was found to be comprehensive, clear, and relevant for patients with acromegaly receiving injectable SSA treatment.

## Introduction

Acromegaly is a rare hormonal disorder where excess growth hormone (GH) and insulin-like growth factor 1 (IGF-1) are produced; it is most often caused by a benign tumor of the pituitary gland [1–3]. As a result of the excess secretion of GH and IGF-1, individuals with acromegaly may experience changes to their facial appearance and enlargement of the hands and feet, as well as active acromegaly symptoms such as headache, sweating, fatigue, soft tissue swelling and joint pain; other comorbidities include sleep apnea, hypertension, diabetes mellitus, bone disease, and cardiovascular disease [4].

Medical therapy is an important option in the acromegaly armamentarium as primary or adjuvant therapy after surgery and/or radiation therapy [5–7]. These therapies fall into three classes: somatostatin analogs, or SSAs (e.g. octreotide, lanreotide, pasireotide), dopamine agonists (bromocriptine and cabergoline), and a GH receptor antagonist (pegvisomant) [3, 8]. SSAs are the most commonly utilized first-line medical therapy for acromegaly patients [9, 10].

SSAs can be administered as either intramuscular (octreotide) or deep subcutaneous injections (lanreotide). Injections are typically given monthly (but can also be prescribed every 6 or 8 weeks) [11, 12] and can be administered either by healthcare professionals in a care setting, or at home by either a healthcare professional or a family member. When receiving injections at home, the majority of patients do not choose to self-inject [13]. Potential side effects associated with SSAs [11, 12] include injection site reactions and gastrointestinal (GI) symptoms (most commonly diarrhea, nausea, and abdominal pain). Some side effects, such as joint pain and headaches, can also be disease-related, and therefore it can be difficult to determine whether they are a result of acromegaly or treatment.

Despite achieving biochemical control on treatment, patients can continue to experience acromegaly symptoms, some of which may worsen towards the end of the injection interval (breakthrough symptoms). Symptoms associated with acromegaly and treatment-related side effects can negatively impact one’s health related quality of life (HRQoL) [14]. Additionally, aspects related to the mode and frequency of treatment administration can impact patients’ lives [15, 16]. Patient-reported outcomes (PROs) are increasingly common as relevant outcomes in studies of acromegaly and its treatment [13, 16–21]. PRO measures have previously been used in studies of acromegaly patients; however, some of these measures (such as the 36-item Short Form Survey [SF-36] [22] or the Treatment Satisfaction Questionnaire for Medication [TSQM] [23]) are not specific to acromegaly, which limits their ability to assess specific aspects of the disease or its treatment. While the Acromegaly Quality of Life Questionnaire (AcroQoL) was developed specifically for use with an acromegaly population [24–26] and asks about physical symptoms and impacts to daily activities and social functioning, it does not address the effectiveness or potential side effects of treatment. A recently developed software tool, the Acromegaly Disease Activity Tool (ACRODAT®) [27], measures disease activity to support clinical decision-making, but like the AcroQoL, it does not address all aspects of acromegaly treatment. The SAGIT® is another recently developed tool designed to assess symptoms, comorbidities, and biochemical aspects of acromegaly [28], but is clinician-reported, intended to assist in clinical practice, and does not assess patient-reported acromegaly- or treatment-related HRQoL. There are no validated PRO measures available that comprehensively assess the impact of treatment and are specifically designed to be self administered by acromegaly patients.

We sought to develop a novel and unique PRO measure, the Acromegaly Treatment Satisfaction Questionnaire (Acro-TSQ) [29], that, once validated, will be applicable for use in clinical trials and clinical practice, together with other clinical, laboratory, and imaging data, to assess and monitor the overall disease and treatment burden of acromegaly. We present here the process used to develop and refine the Acro-TSQ.

## Methods

**Fig. 1:**
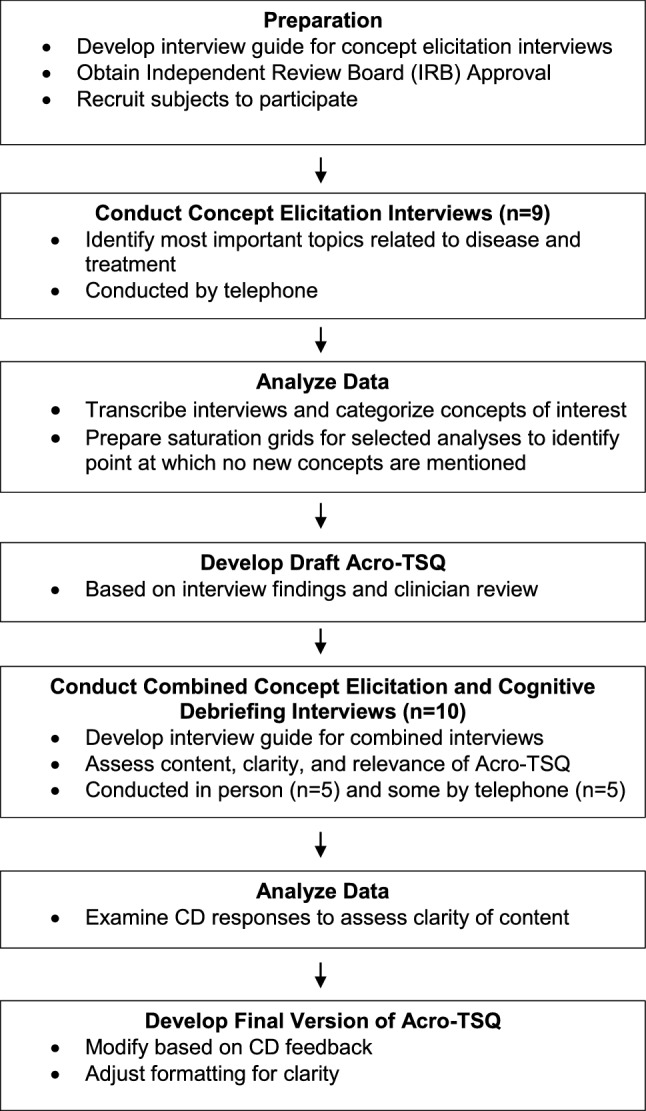
Development process of the Acro-TSQ

The development of the Acro-TSQ involved several steps (Fig. 1) to ensure that the questionnaire addressed what patients considered to be the most relevant aspects of acromegaly and its treatment, and that the questions were clear and comprehensive. To begin, a literature review was conducted to determine whether there were any suitable acromegaly-specific patient reported outcome (PRO) measures that could be utilized in upcoming clinical trials. No acromegaly-specific measures were identified that specifically assessed concepts such as patient perceptions of the efficacy of acromegaly treatment, acromegaly symptoms, side effects, injection site reactions, and convenience and satisfaction with treatment. During the development process, the team was cognizant of the need to address both acromegaly symptoms and the impact of treatment (effectiveness, adverse reactions, etc.) on the patient experience. Concept elicitation (CE) interviews and (later) combined CE and cognitive debriefing (CE/CD) interviews were conducted to solicit information that would assist in drafting questions and refining them to ensure the tool’s content, clarity, and relevance. Soliciting patient input during the development and refinement of a PRO measure is recommended by the Food and Drug Administration (FDA) in a published document containing guidelines for PRO development [30]. The goal of CE interviews is to elicit issues that are most important and relevant to a target population [31]. CD interviews allow patients to evaluate drafts of a measure to assess understanding and obtain feedback on content, format, recall period, and response options [32]. The Acro-TSQ development also involved input by clinical experts, including an outside endocrinologist, project team members as well as all co-authors.

### Subject identification

A convenience sample of acromegaly patients was recruited from the Acromegaly Community, Inc. (www.acromegalycommunity.org), using an IRB-approved advertisement posted on its Facebook page. Those eligible for inclusion were aged 18 to 75 years of age with a diagnosis of acromegaly by documented evidence of a GH-secreting pituitary tumor currently receiving parenteral SSAs (octreotide or lanreotide) at a stable dose for at least 6 months. To the extent possible, individuals were enrolled taking into account demographic diversity. Subjects also signed a Medical Release Form that allowed their treating endocrinologist to provide clinical information (IGF-1 values, time since diagnosis, previous treatments, comorbid conditions) on their behalf; in instances when the physician did not respond after repeated inquiries, subjects were asked to self-report clinical information.

### Initial CE interviews

The goal of the CE interviews was to elicit important concepts from subjects regarding acromegaly and its treatment. Using information gathered from the published literature [23–25, 33–35], a semi-structured interview guide was developed specifically for this study focusing on acromegaly symptoms and treatment-related issues. At the start of the interview, interviewers explained to respondents that the interviews were being conducting to learn more about how they felt about their acromegaly treatment. Interview questions asked about treatment effectiveness, patient experience, and the emotional impact of treatment. Questions were intentionally designed to be open-ended, but the interview guide also included probes for the interviewer to use when necessary. For example, subjects were asked “Thinking about [insert current treatment], how well do you think it works? Why?” but interviewers could use additional questions to obtain more information: “Tell me more about the pattern of your symptoms. Which symptoms do you start experiencing? (Probes: joint or muscle pain, sweating, fatigue, headaches, swelling of extremities, intolerance of heat, etc.)” When asking about discomfort with treatment, if subjects indicated that they’d had an injection site reaction, the interviewer responded with, “Tell me about that. (Probes: how did it feel? How long did it last? What did it look like?)” Additional examples of interview questions are presented in Table 1A. Using the most commonly-reported concepts from these interviews and, whenever possible, terminology that subjects used when describing an issue, an initial draft of the Acro-TSQ was developed and reviewed by an interdisciplinary team, including endocrinologists. The draft Acro-TSQ focused on symptoms, effectiveness, burden, and side effects of treatment, and route of administration, including the potential use of an oral SSA.

**Table 1:** Example questions from (A) interview guides and (B) Acro-TSQ

(A) From interview guides	
Topic area	Question
*Concept elicitation interviews*	
Treatment effectiveness	“Thinking about [insert current treatment], how well do you think it works? Why? Tell me more about the pattern of your symptoms.” If necessary, probe symptoms: joint or muscle pain, sweating, fatigue, headaches, swelling of extremities. “Tell me more about each symptom.” “Do you ever have symptoms even though you are receiving treatment?”
Treatment experience	“How much discomfort, if any, do you experience with your treatment? Tell me more about that. (If an injection) have you ever had an injection site reaction? [If yes] Tell me about that. (Probes: how did it feel? how long did it last? what did it look like? do you experience pain, lumps/nodules, swelling, inflammation/infection, bruising/hematoma, irritation (red skin, itching), other skin lesions (pitting, abscess, discoloration, ulcer))?” “Have you ever experienced any side effects from this treatment? [If yes] What side effects have you experienced? (Probes: nausea, diarrhea, abdominal pain, constipation, fever, dizziness, headaches, fatigue, flatulence, other skin lesions (pitting, abscess, discoloration, ulcer)”
Emotional impact	“How much, if at all, does your current treatment affect you emotionally? (Probes: feeling angry, frustrated, dependent, anxious, fearful, stressed, loss of independence) Why? How much does your treatment interfere with your ability to socialize? (Probes: spend time with family or friends) Why?”
*Cognitive debrief interviews*	
Clarity	“Looking at question [insert question] in your own words, what is this question asking you?” “Do you have any suggestions about how this question could be revised? If yes, please describe. Why do you think that is clearer?”
Format	“Was it helpful or unhelpful to have the words in the parentheses? Why? Are there other examples that you recommend we include in the parentheses or any that you recommend we omit? If yes, please describe.” “Do you think we need more or fewer response options for any of the questions? Why? Do you have any suggestions for a different set of response options for any of the questions? If yes, why do you prefer these response options?”
Recall period	“What timeframe were you thinking about when you answered the questions that were asking you to think ‘in general’? Was it confusing that the timeframe here was ‘in general’ and the questions right before this used the past 4 weeks? Why or why not?” “Was it easy or difficult to think about ‘the past 4 weeks’?”

(B) From Acro-TSQ after debriefing	
Domain	Question
Treatment effectiveness	“**In the past 4 weeks**, how would you rate the ability of your current acromegaly treatment **to improve your acromegaly symptoms**?” “**In the past 4 weeks**, even though you were being treated with medication for acromegaly, did you ever experience acromegaly symptoms?”
Symptom burden	“**In the past 4 weeks**, how bothered were you, if at all, by the **amount of time** you experienced acromegaly symptoms?” “**In the past 4 weeks**, how much, if at all, have **acromegaly symptoms interfered with your ability to do daily activities (e.g., walking or moving about, going up/down stairs, household chores, errands, cooking, taking care of children or grandchildren, etc.)**?”
Treatment side-effects	“**In the past 4 weeks**, how much, if at all, have treatment-related **gastrointestinal side effects interfered with your ability to participate in leisure activities (e.g., going to restaurants or movies, watching sports, exercising, spending time with family and friends, etc.)**?” “**Since your last injection**, how bothered, if at all, were you by treatment-related **injection site reactions during the FIRST FEW DAYS after each injection**?”
Convenience of treatment	“How convenient or inconvenient is it to have your current acromegaly treatment **administered in its current form (e.g., injections)**?” “How bothered, if at all, were you with having to **travel to the doctor’s office or clinic to receive your injections?**”
Overall satisfaction	“**In the past 4 weeks**, how **satisfied or dissatisfied** have you been with your current acromegaly treatment?”

### Combined CE/CD interviews

After the initial draft of the Acro-TSQ was developed, combined CE/CD interviews were conducted. The goal of these interviews was two-fold: as with the CE-only interviews, one goal was to elicit important concepts of acromegaly and its treatment from respondents using a subset of the questions from the CE interview guide. Conducting interviews using a combined approach allowed us to maximize the information we collected from each individual. Additionally, the CD portion of these interviews was intended to assess how well respondents understood questions included in the draft Acro-TSQ and obtain feedback on its content, format, recall period, and response options. Subjects were asked to re-state questions in their own words and provide feedback on their clarity. For example, they were asked “What is this question asking you? Do you have any suggestions about how this question could be revised? If yes, please describe. Why do you think that is clearer?” When asked about parenthetical information provided in questions and about response options, subjects were given the opportunity to suggest alternatives and explain why they preferred those alternatives (Table 1A). These interviews involved a combination of in-home and telephone interviews and were conducted using a semi-structured interview guide developed specifically for this study. (Note: The Interview Guides are available upon request from the corresponding author.) The Acro-TSQ was updated iteratively as interviews were conducted.

### Analysis

All interviews were recorded and transcribed, and interview data were coded using MAXQDA (Verbi GmbH, Berlin, Germany), a qualitative data analysis software to organize and categorize the concepts of interest. The analysis of interview data was conducted using a thematic approach that included a search for themes as well as specific codes within each theme. Specifically, the coder identified patterns or themes within the transcript and categorized the data into these themes. Specific codes were identified within each theme.

Each transcript was coded by one coder, then reviewed, summarized, and analyzed by a second coder. Saturation grids were prepared to determine the point at which no new concepts were mentioned by subsequent subjects. Grids were prepared at the end of the CE only interviews and again once the combined CE/CD interviews were conducted.

Human subjects’ research approval for study was provided by an independent, scientific review committee, The Copernicus Group, Cary, NC on April 1, 2015.

## Results

### Demographic and clinical characteristics

A total of 19 subjects completed interviews. Nine CE-only interviews, all done by telephone, and 10 combined CE/CD interviews (5 in person, 5 over telephone) were conducted. The demographic and clinical characteristics of the subjects are summarized in Table 2 (clinical data were not available for one subject). The majority of subjects was female (58%), Caucasian (79%), and worked for pay (63%). The average age was 47 years old. All participants resided in the US.

**Table 2:** Demographic and clinical characteristics

	CE	CE/CD	Total
	(n = 9)	(n = 10^a^)	(n = 19)
Age, years (mean ± SD) (range)	44 ± 9.8 (31–57)	50 ± 14.4 (31–84)	47 ± 12.5 (31–84)
Gender, n (%)			
Male	4 (44%)	4 (40%)	8 (43%)
Female	5 (56%)	6 (60%)	11 (58%)
Education, n (%)			
Less than HS	0	0	0
HS diploma	2 (22%)	3 (30%)	5 (26%)
Some college	3 (33%)	2 (20%)	5 (26%)
College degree	2 (22%)	3 (30%)	5 (26%)
Professional or advanced degree	2 (22%)	2 (20%)	4 (21%)
Ethnicity, n (%)			
Caucasian	8 (89%)	7 (70%)	15 (79%)
African American	0	3 (30%)	3 (16%)
Latino Hispanic	1 (11%)	0	1 (5%)
Marital status, n (%)			
Married	6 (67%)	7 (70%)	13 (68%)
Living with partner	0	0	0
Widowed/divorced/separated	1 (11%)	2 (20%)	3 (16%)
Single, never married	2 (22%)	1 (10%)	3 (16%)
Household Income, n (%)			
< $25,000	1 (11%)	3 (30%)	4 (21%)
$25,000–$49,999	2 (22%)	1 (10%)	3 (16%)
$50,000–$74,999	3 (33%)	3 (30%)	6 (32%)
$75,000–$99,999	3 (33%)	2 (20%)	5 (26%)
≥ $100,000	0	1 (10%)	1 (5%)
Work status, n (%)			
Full time for pay	3 (33%)	4 (40%)	7 (37%)
Part time for pay	1 (11%)	4 (40%)	5 (26%)
Don’t work for pay due to acromegaly	3 (33%)	2 (20%)	5 (26%)
Don’t work for pay, not due to acromegaly	2 (22%)	0	2 (11%)
Time since diagnosis, year (mean ± SD) (range)	10.0 ± 9.2 (0–32)	6.9 ± 5.0 (1–15)	9.0 ± 7.4 (0–32)
Surgery to treat acromegaly			
N	9	9	18
Ever received (n, %)	9 (100%)	8 (89%)	17 (94%)
Radiation to treat acromegaly			
N	9	9	18
Ever received (n, %)	6 (67%)	1 (11%)	7 (39%)
Current treatment			
Lanreoltide (Somatuline Depot/Autogel)	7 (78%)	8 (80%)	15 (79%)
Octreotide (Sandostatin LAR Depot)	2 (22%)	2 (20%)	4 (21%)
Rating of acromegaly control			
Controlled	8 (89%)	5 (55%)	13 (72%)
Partially controlled	1 (11%)	3 (33%)	4 (22%)
Not well controlled	0	1 (11%)	1 (6%)
Co-morbid conditions (ever)			
Hypertension	5 (44%)	4 (44%)	9 (50%)
Diabetes	3 (33%)	6 (66%)	9 (50%)
Sleep apnea	3 (33%)	4 (44%)	7 (39%)
Headaches	4 (44%)	5 (55%)	9 (50%)
Osteoarthritis	3 (33%)	4 (44%)	5 (28%)
Depression	2 (22%)	6 (66%)	8 (44%)
Hypopituitarism	5 (55%)	1 (11%)	6 (33%)
Gallstones	1 (11%)	3 (33%)	4 (22%)
GI issues	1 (11%)	4 (44%)	5 (28%)
Other	3 (33%)	4 (44%)	7 (39%)

^a^One subject in the CE only group, and six subjects in the combined CE/CD group self-reported their clinical data since their endocrinologist was not willing or not able to provide HOS with the clinical data. Clinical data are not available for one subject in this group

The mean (SD) time since diagnosis was 9.0 (7.4) years, and the self-reported level of acromegaly control was rated as “controlled” by 72% (13/18) of subjects, “partially controlled” for 22%, and “not well controlled” for 6%. The majority of subjects (94%, 17/18) had received surgery at some point to treat their acromegaly. Fifteen (79%) subjects were currently receiving lanreotide (Somatuline Depot/Autogel) and 4 (21%) were currently receiving octreotide LAR (Sandostatin LAR Depot). Eight subjects indicated that they self-inject their treatment.

### Results of the CE interviews

Although a total of 19 subjects participated in either CE or CE/CD interviews, not all 19 subjects responded to all questions. This was due to a variety of reasons, including the fact that not all the questions from the initial CE interviews were included in the CE/CD interviews in the interest of time. Additionally, some questions were only asked of subjects who responded affirmatively to a previous question. For example, only subjects who reported experiencing breakthrough symptoms were asked to list which breakthrough symptoms they experienced and whether they impacted their ability to perform daily activities. In addition, we could not exceed the allotted time of 60 minutes for each interview, as designated in the study protocol.

#### Treatment experience, including breakthrough symptoms and injection-site reactions

**Fig. 2:**
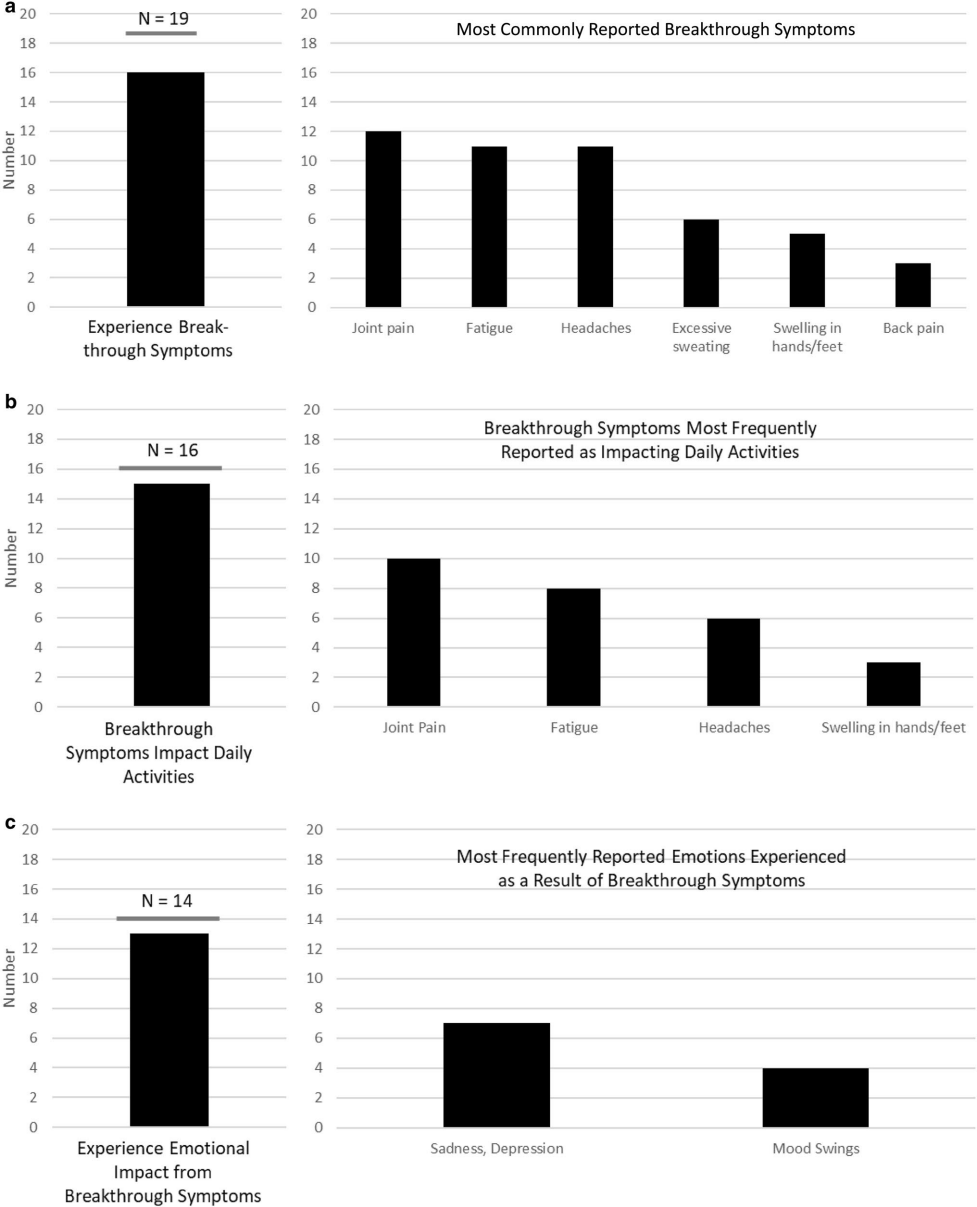
Impact of acromegaly

Most subjects reported experiencing breakthrough symptoms while on treatment, most commonly joint pain, fatigue, and headaches (Fig. 2a). These symptoms occurred throughout the injection cycle or in the days or the week prior to the next scheduled injection. Of subjects who indicated that they experienced breakthrough symptoms, almost all reported that these symptoms impacted their ability to do daily activities (Fig. 2b). Symptoms cited as being the most impactful included: joint pain, fatigue, and headaches. It was also common for subjects to report that they experienced an emotional impact associated with their acromegaly symptoms (Fig. 2c), with some indicating that they feel “a real bad depression” as well as “frustration and anger.”

**Fig. 3:**
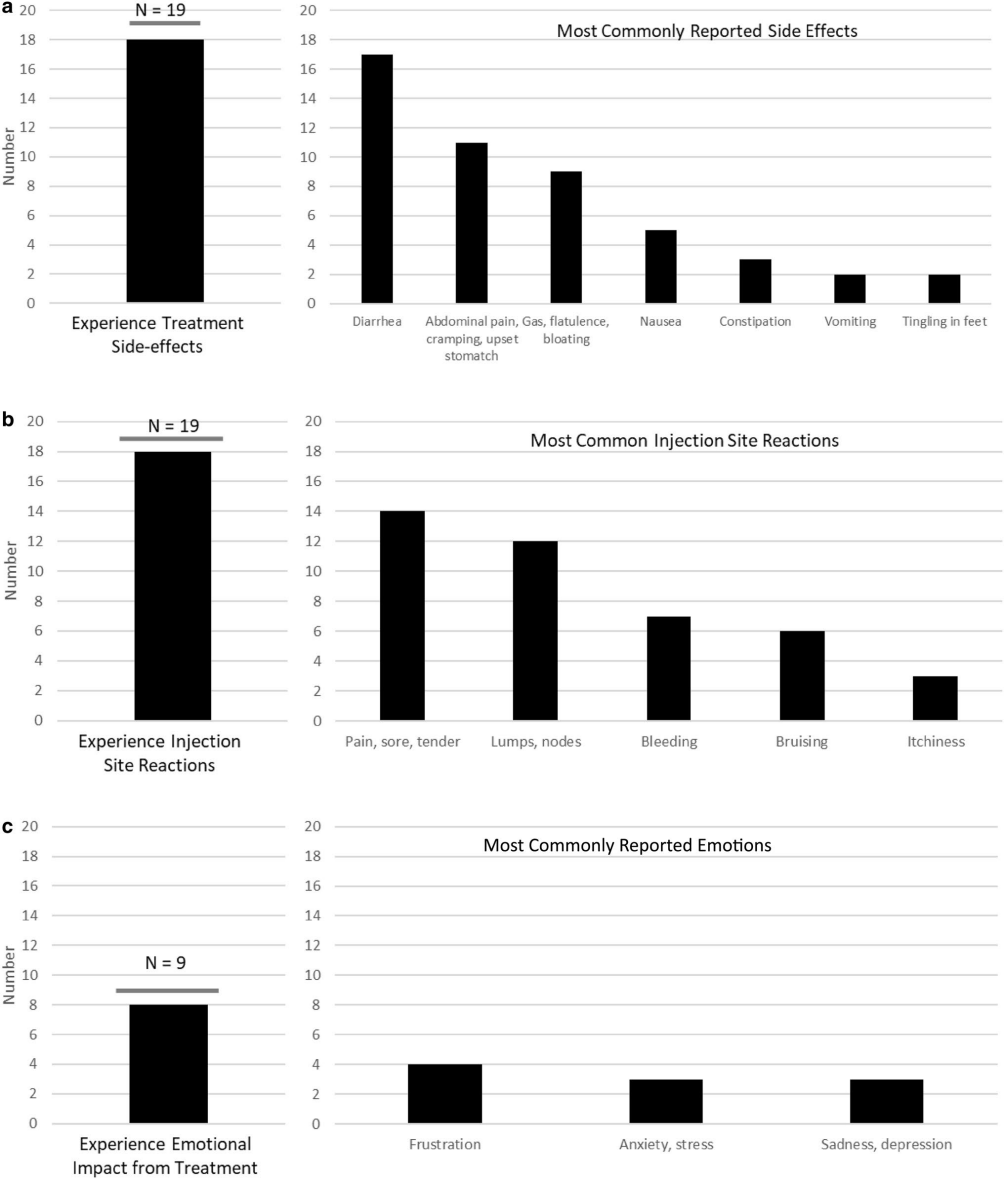
Impact of Aromegaly Treatment

When asked about discomfort and injection-site reactions (Fig. 3b), all but one subject reported experiencing signs/symptoms at the injection site. Respondents frequently mentioned pain or discomfort, although the reported duration and severity varied. One subject indicated that “it’s just a few seconds,” while another said that the discomfort lasted “for maybe a day or two.” Other common injection-site reactions mentioned included lumps/nodules, bleeding, and bruising. Around half reported that these adverse reactions make them anxious about future injections, but most indicated that they did not impact their daily activities, although some reported that “the rest of the day is kind of shot” or that they “don’t plan anything” and “just pretty much will hang around the house.” It was common for subjects to experience treatment-related side effects (Fig. 3a), such as diarrhea, abdominal pain, and nausea; a few also experienced constipation, vomiting, and tingling in their feet. Most reported that these side effects impacted their ability to do daily activities. However, for some, the side effects were short-lived; one respondent said, “…although after the injection I’m sick for two to three days, the rest of the month I feel pretty good.”

#### Impact on functioning

Most respondents reported negative impacts of treatment on their emotional and social functioning (Fig. 3c, Table 3), citing anxiety due to the anticipation of the pain and side-effects (“the actual injection causes me quite a bit of anxiety now so emotionally it takes a toll a few days beforehand when I am preparing myself” and “it does create anxiety and worry for me about the side-effects”), or hope for a different treatment modality (“my hope is that there’s a pill or something that’s more effective with the same amount of side-effects or less”).

**Table 3:** Representative quotes from concept elicitation interviews and combined concept elicitation and cognitive debriefing interviews

Topic area	Representative quotes
Effectiveness of current treatment	“It’s clear that the [treatment] is certainly working and I’m certainly seeing results because in the past when we have just attempted to go off of that medication even for a month, you can see drastically that those levels shoot right back up and not just a little bit. It’s usually a pretty significant increase in a short amount of time” “Well it seems to be controlling my levels. For one, it’s a one-time injection and although after the injection I’m sick for two to three days, the rest of the month I feel pretty good” “Well I think, why I don’t think it’s symptom wise is working very well is because I still experience the same, the sweats, like I get very hot, I get very anxious and these are ongoing where I’m on [treatment] or not” “Well, it doesn’t have quite, as far as I understand it doesn’t have quite the effect on this as some of the other treatments and the side-effects that it does have is significant for me so I’m getting a lot of side-effects on a medium level dose that’s, at best, moderately effective in keeping my IGF-1 in check”
Discomfort or reactions to injections	“It’s painful, it certainly is painful and the act of actually sticking it is, it hurts. However, it’s just a few seconds though” “It depends on the month, but on average mild discomfort for maybe a day or two, just injection site pain” “I get a lump, a little marble sized lump, but it doesn’t bother me” “No redness. A little bruising once in while if we hit a blood vessel” “Well when I first get it, it’s burning, it’s a burning feeling and it’s, it probably stays sore for that first day where it’s uncomfortable. So I don’t try, I try not to schedule a lot to do that day” “The itching probably lasts for a couple of days. The swelling lasts probably for about, I’d say about a week. The redness kind of goes away more of when the itching goes away”
Impact of treatment on emotional and social functioning	“The actual injection causes me quite a bit of anxiety now so emotionally it takes it tolls a few days beforehand when I am preparing myself, the fact that I have to schedule the appointment and go to the hospital, I don’t know, every time I have to go to the hospital it’s just a reminder, I mean that’s where I had my surgery, it’s where sick people go so it’s a constant reminder, going to the hospital, going in there that I’m sick and I have to keep coming here and I have to keep seeing a nurse” “It’s not just anxiety. It’s a fear of the future. In terms of, it does create anxiety and worry for me about the side-effects. I mean at this point after 24 or 26 or 28 shots the side-effects should be fairly predictable, but again I worry about that particularly if the disease process returns and my hope is that there’s a pill or something that’s more effective with the same amount of side-effects or less”
Satisfaction with acromegaly treatment (on a scale from 0 to 10, where 0 is not at all satisfied and 10 is extremely satisfied)	“Right now I would give it like a two or a three. If you talked to me three months ago I would have given it a zero, but I’ll give it a two or three right now just because it’s actually doing something. It took four years to do something” “I’d say 8. Because it works, it keeps the symptoms down and all” “I would say probably a nine. I’m satisfied with it and it helps and everything. The only two issues I have is the wear-off time and then the pain of the needle, but like I said I don’t really experience that because I have the medication that numbs it, but I still get anxiety about it” “I’m not really satisfied, maybe a five”

#### Treatment satisfaction

When asked how well their current therapy worked, most subjects answered positively, saying that their treatment “works really well,” or that it is “certainly working” and “seems to be controlling my levels,” although some were not completely satisfied as evidenced by statements like, “I don’t think it’s… working very well because I still experience the same [symptoms]” and “at best, [the treatment is] moderately effective in keeping my IGF-1 in check” (representative quotes are included in Table 3). Subjects were asked to rate their satisfaction with their acromegaly treatment on a scale ranging from 0 to 10, where 0 is “not at all satisfied” and 10 is “extremely satisfied.” The mean satisfaction rating was 6.8, although a range of responses was provided. Some respondents’ statements reflected low levels of satisfaction, such as, “Right now I would give it like a two or a three,” and “I’m not really satisfied, maybe a five.” Others offered very positive statements, like, “I’d say 8. Because it works, it keeps the symptoms down and all,” and “I would say probably a nine. I’m satisfied with it and it helps and everything.”

#### Development of the Acro-TSQ

Based on the literature review and initial CE interviews, a draft version of the Acro-TSQ containing 29 items was developed and incorporated commonly reported concepts. For example, eight of the initial nine subjects reported experiencing pain and lumps/nodules from the injections. Therefore, these symptoms were included to describe injection site reactions. In addition, transcripts were reviewed and, to the extent possible, language from the subjects was utilized to develop the items. Input was also received from clinicians on the draft measure to ensure clinical relevance and to help identify any potential missing concepts. The questionnaire was then reviewed with each subject during the subsequent combined CE/CD interviews.

#### Results of CD interviews

Information from the CD interviews was obtained from the 10 combined CE/CD interviews (5 in person, 5 over telephone). All subjects found the measure to be relevant, easy to complete and found the response options to be clear; 67–100% of subjects were able to correctly paraphrase each item. Representative quotes from these interviews appear in Table 3.

When asked if it was easy or difficult to think about the recall periods (e.g., “the past 4 weeks”), most thought the recall periods were appropriate and easy to think about.

All subjects thought all items were relevant and that the questionnaire was easy to complete, and all but one thought the order of the questions was appropriate. On average, it took 16 min to complete the questionnaire (n = 8: range: 10–30 min). Changes to the Acro-TSQ as a result of the debriefing feedback included minor wording or formatting changes or adding more examples of symptoms or activities to clarify questions for respondents. For example, when asking about the occurrence of treatment-related GI side effects, the original version listed “abdominal pain, diarrhea, or nausea” as examples; the final version also included “vomiting, constipation, gas, or acid reflux.” In another example, multiple subjects indicated that they experienced some symptoms “throughout the month” but that “those same symptoms and possibly other symptoms get worse before the next dose” so response options were added to allow for these types of situations.

#### Take-aways from CE and CE/CD interviews and resulting version of Acro-TSQ

The resulting version of the Acro-TSQ, after incorporating the feedback, contains 26 items which covers symptoms and symptom control, GI-related side effects and their impact on daily activities, the emotional impact of treatment, convenience and ease of use, and overall satisfaction (Table 1B). Some questions ask the respondent to rate aspects of treatment or the impact of side effects. When assessing emotions, subjects are asked to rate (from 0 “did not have” to 10 “worst imaginable”) how much they felt sad, anxious, frustrated/angry, or dependent on others. To assess how convenient or easy treatment is to use, subjects are asked about the impact of treatment on their ability to make plans with friends or to travel, and the level of bother with having to schedule injections. Most questions have a recall period of the past 4 weeks, although some questions inquire about aspects “in general.”

## Discussion

PROs are increasingly recognized as important metrics for evaluating treatment effectiveness (symptomatic control), as well as treatment side effects and convenience [13, 17, 18]. Evaluation of HRQoL can highlight the patient’s viewpoint of clinical aspects not always discussed at the visits with providers, but is of significant importance for the patient’s daily life. Treatment often focuses on normalizing GH and/or IGF-1 levels [10], but studies have failed to demonstrate a consistent correlation between biochemical control and HRQoL [13, 26]. The impact of acromegaly treatment (mode of administration, convenience, effectiveness, and side-effects) itself on HRQoL is not well evaluated by currently available tools. Therefore, a PRO measure that allows clinicians and researchers to assess patient well-being, symptom interference, and the impact of treatment reflects the most comprehensive assessment of patients’ experiences with acromegaly and its treatment.

The Acro-TSQ was developed based on input from patients with acromegaly currently receiving SSA treatment and was reviewed by endocrinologists; its elements include aspects of the disease and treatment that patients consider to be important, relevant, and impactful. The interviews with acromegaly patients revealed that most feel that their current treatment is effective in reducing symptoms, and many are very satisfied overall. The interviews also identified several key concepts that may negatively impact patients’ HRQoL, including that many patients receiving SSA treatment continue to experience breakthrough symptoms and that there are significant adverse outcomes associated with treatment, including side-effects and emotional and social repercussions. Aspects of treatment which impact satisfaction include the frequency and location of administration, the ability to plan life around injections, ease of scheduling appointments, costs, and side-effects (injection-site reactions, GI symptoms).

Information gathered from the CE and CD interviews was relatively similar to results reported in other recent studies. In a multi-center study of 195 patients (Germany, the UK, and the Netherlands) receiving either octreotide or lanreotide injections, Strasburger et al. used an endocrinologist-developed questionnaire to obtain patient-reported data regarding their experience with and the emotional burden of both symptoms and treatment. Many of the most commonly reported symptoms (joint pain, fatigue, snoring, excessive sweating, and headaches) mirror those reported in the current study [13]; subjects in both studies also reported pain, lumps/nodules, and bruising at the injection site and feeling burdened by treatment injections [13]. In a separate study of 59 patients receiving lanreotide injections described by Salvatori et al., the reported treatment-related adverse events reported were similar to those reported during the current study, but fewer reported injection site pain, irritation, or pruritus [36]. Subjects were not asked about the emotional or psychological impacts of acromegaly symptoms or treatment [36]. While some available PROs provide similar kinds of information regarding the frequency of events associated with acromegaly symptoms and treatment, the Acro-TSQ incorporates feedback solicited from patients on whether the measure includes relevant or impactful aspects of the disease or treatment. In addition, the development process for the Acro-TSQ followed practices recommended by the FDA guidance document for PRO development [30].

The Acro-TSQ was found to be comprehensive, clear, and relevant; minor revisions were made to certain questions to improve clarity or for emphasis. The results in this study confirm the burden of injectable treatment and highlight the importance of collecting PRO data.

Both CE and CD interviews (conducted both in person and over the phone) were used to solicit patient input during development, as recommended by FDA guidance. However, there are several limitations of this study. First, as with most qualitative research studies, the number of patients participating is small. Second, subjects were selected from a patient online community in which they have chosen to participate, therefore may not reflect the real-world population. Further, some clinical data were obtained via self-report because the patient’s clinician was unresponsive to requests to provide patient information. Lastly, due to the length of the interviews, not all questions were asked of all subjects, further limiting the sample size of some responses.

In conclusion, the Acro-TSQ is a novel comprehensive, clear tool found to be relevant for patients with acromegaly receiving SSA treatment. It was developed using both concept elicitation and cognitive debriefing interviews as recommended by the FDA, and represents the first PRO measure available to assess treatment-related aspects specifically for patients treated for acromegaly. Once validated, it will allow clinicians and researchers to consider patient experiences of acromegaly and satisfaction with treatment. The measurement properties (including internal consistency and test–retest reliability, construct and known groups validity, and responsiveness) of the Acro-TSQ are currently being validated in a larger study. The Acro-TSQ will be available upon request once the validation analyses are completed and the questionnaire is finalized.
